# Herpes Simplex Virus Type 2 Screening in Persons with and Without HIV: Evidence, Challenges, and Future Directions

**DOI:** 10.1007/s11904-026-00771-6

**Published:** 2026-02-02

**Authors:** Laith J. Abu-Raddad, Hiam Chemaitelly, Anna Wald, Christine Johnston

**Affiliations:** 1 Infectious Disease Epidemiology Group, Weill Cornell Medicine-Qatar, Cornell University, Doha, Qatar; 2 Department of Population Health Sciences, Weill Cornell Medicine, Cornell University, New York, NY USA; 3 Department of Public Health, College of Health Sciences, QU Health, Qatar University, Doha, Qatar; 4 College of Health and Life Sciences, Hamad bin Khalifa University, Doha, Qatar; 5 Department of Medicine, School of Medicine, University of Washington, Seattle, WA USA; 6 Department of Epidemiology, University of Washington, Seattle, WA USA; 7 Department of Laboratory Medicine and Pathology, University of Washington School of Medicine, Seattle, WA USA; 8 Vaccine and Infectious Disease Division, Fred Hutchinson Cancer Center, Seattle, WA USA

**Keywords:** HSV-2, Genital herpes, Screening, HSV-1, Diagnostics, Public health

## Abstract

**Purpose of Review:**

Herpes simplex virus type 2 (HSV-2) infection is one of the most prevalent sexually transmitted infections worldwide, with implications for HIV acquisition, transmission, and disease progression. This review synthesizes current evidence and guidance on HSV-2 serologic screening, emphasizing its relevance for HIV prevention and care.

**Recent Findings:**

International guidelines advise against routine general population-level serologic screening for HSV-2 in asymptomatic persons. Key limitations include poor test specificity, the absence of potent antivirals or therapeutic vaccines, lack of curative therapy, no demonstrated population-level benefit, and psychosocial harms associated with diagnosis. Current practice instead emphasizes diagnostic testing in symptomatic persons and targeted screening in defined contexts—such as among people with HIV in specific clinical situations, sex partners of those with HSV-2 infection, certain pregnant women, persons seeking sexual health care, and persons with recurrent or atypical symptoms—where results may directly inform management. Emerging technologies, including highly specific assays, novel potent antivirals, therapeutic vaccines, and curative strategies, may eventually shift the cost–benefit balance of general screening.

**Summary:**

Evidence supports targeted rather than general population-level screening to maximize clinical benefit while minimizing harm. New evidence demonstrating that interventions can achieve measurable population-level reductions in disease burden or transmission, together with future advances in diagnostics and therapeutics, may eventually justify integrating routine HSV-2 screening into broader contexts, including into HIV prevention and care.

## Introduction

Herpes simplex virus type 2 (HSV-2) infection is a common, chronic sexually transmitted infection (STI) that contributes substantially to global morbidity and health economic burden [1–5]. Most infections are asymptomatic or unrecognized, yet the infection is characterized by frequent subclinical viral shedding and, in a subset of persons, recurrent symptomatic disease [6–10]. HSV-2 is the leading cause of ulcerative genital herpes and the most common cause of genital ulcer disease worldwide [11–16]. The infection has well-established biological and epidemiological links with HIV infection [17–19] and has contributed to the spread of HIV globally [18, 20, 21].

Beyond the clinical burden of genital disease, HSV-2 infection imposes social and psychological costs, adversely affecting sexual relationships, quality of life, and mental health, with increased risks of depression, anxiety, and low self-esteem [22–28]. Although uncommon, transmission from women with genital HSV during delivery can result in neonatal herpes—a severe, often life-threatening condition with high mortality and long-term morbidity [29–32]. In response to this multifaceted disease burden, the World Health Organization (WHO) and the HSV Addendum to the Sexually Transmitted Infections National Strategic Plan for the United States: 2021–2025 have called for intensified efforts to reduce HSV-related disease, highlighting the need for improved prevention and treatment strategies, including the development of effective vaccines [33–36].

These considerations frame the discussion on HSV-2 screening. Detecting asymptomatic infections could, in theory, enable targeted counseling and antiviral therapy to reduce transmission and improve clinical management, including among persons with both HIV and HSV-2 and with HSV-2–discordant partners. However, the suboptimal specificity of available serologic assays—with a high proportion of false positives—the absence of curative therapies or highly effective antivirals capable of extensively reducing HSV-2 transmission or mitigating HIV acquisition and transmission, the lack of therapeutic vaccines, and the psychosocial burden associated with an HSV-2 diagnosis collectively limit the rationale for population-level screening.

This review first outlines the biology, epidemiology, clinical burden, links to HIV, economic impact, and diagnostic methods for HSV-2, providing the foundation for a critical examination of the benefits and harms of screening. Screening can be classified as general population-level, involving routine testing of asymptomatic persons regardless of risk, or targeted, focusing on groups at elevated risk or within specific contexts where results can inform case management. In contrast, diagnostic testing refers to the testing of persons with unconfirmed clinical diagnoses of genital herpes or those presenting with signs or symptoms suggestive of infection, where results directly guide case management.

In current practice, HSV-2 testing primarily serves as a combination of diagnostic testing (for persons with symptoms but no confirmed diagnosis) and targeted screening (for partners of persons with HSV-2 or those at higher risk, such as people with HIV, pregnant women, or those seeking sexual health care) [37–45]. General population-level screening, however, is neither recommended nor supported by current guidelines or available evidence [37–45].

This review also examines the public health implications of these approaches and explores emerging advances in therapeutics, vaccines, and diagnostics that could shift the balance of evidence for or against broader implementation of HSV-2 screening.

## Natural History and Transmission of HSV-2

HSV-2 infection follows a complex and dynamic natural history [6–8]. After sexual acquisition, the virus establishes lifelong latency in the sacral ganglia of sensory nerves, with periodic reactivation [7, 8]. Clinical expression is highly variable: some persons may remain asymptomatic or have unrecognized infection, whereas others experience recurrent episodes of urogenital symptoms which may or may not include ulcerative genital lesions [6–10]. Recurrences vary in frequency, duration, and severity, ranging from mild and infrequent to persistent and debilitating disease [6, 9, 10, 46–50]. Among immunocompetent persons, the predictors of the frequency and clinical severity of reactivations remain poorly understood [6, 7, 51]. Effective immune control limits viral reactivation and subsequent shedding, whereas immune compromise—such as in untreated people with HIV—is associated with more frequent, prolonged, and severe recurrences [52–54].

Most HSV-2 infections are unrecognized and undiagnosed [9, 10, 48], and this diagnostic gap hampers infection control efforts. Educational interventions to improve symptom recognition have shown measurable benefits, with most HSV-2–antibody seropositive persons subsequently able to identify or recall typical episodes of infection once counseled [9, 10, 48]. However, even in the absence of symptoms, HSV-2 can be intermittently shed from the genital mucosa [6, 7], sustaining an unrecognized reservoir of persons who may transmit the virus to sex partners or neonates.

The majority of transmissions occur from persons unaware of their infection and without lesions at the time of contact [55–57]. This combination of high prevalence of unrecognized infection, subclinical viral shedding, and transmission in the absence of symptoms limits the effectiveness of symptom-based prevention and underscores the potential role of screening to identify those with otherwise undiagnosed infections, enabling targeted counselling, education, and, where indicated, antiviral suppression.

### Global and Regional Epidemiology of HSV-2

**Fig. 1:**
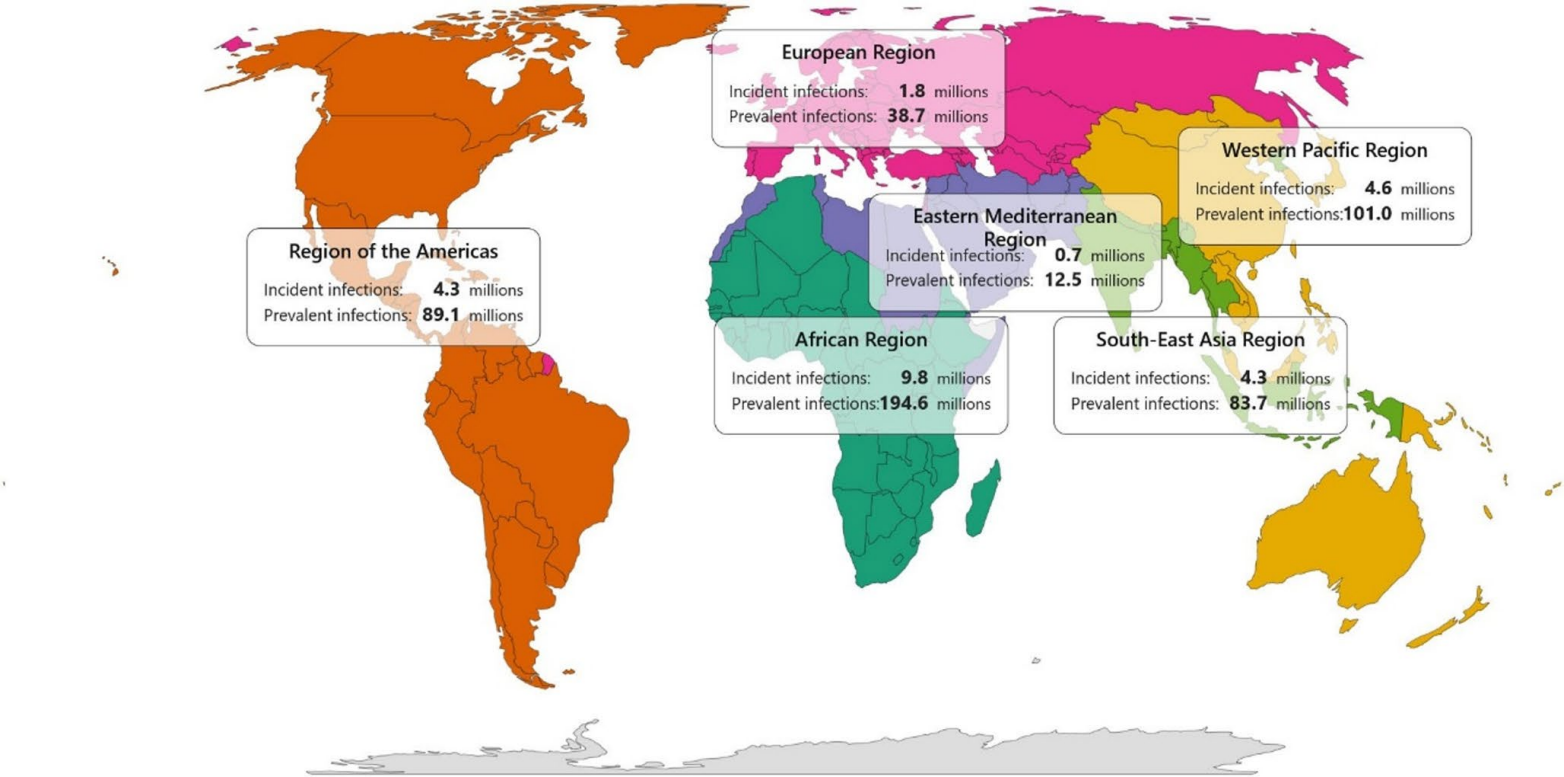
Global distribution of annual incident and prevalent HSV-2 infections by World Health Organization region, 2020 [1]. Incident infections denote new cases within the year; prevalent infections denote the number of people living with HSV-2 infection. Abbreviations: HSV-2, herpes simplex virus type 2

In 2020, an estimated 25.6 million people aged 15–49 years acquired new HSV-2 infections globally (Fig. 1) [1]. In the same age group, 519.5 million people were living with infection, corresponding to a global prevalence of 13.3% [1]. Sub-Saharan Africa bore the greatest burden, accounting for nearly 10 million new infections—38% of the global total—and having both the highest prevalence and the largest absolute number of people living with infection [1].

HSV-2 prevalence varies substantially by region. The highest prevalence is observed in sub-Saharan Africa (37.3%) [11], followed by Latin America and the Caribbean (21.6%) [15], North America and Australia/New Zealand (approximately 15%) [14, 57, 58], Europe (12.4%) [13], Asia (12.1%) [12], and the Middle East and North Africa (5.1%) [59].

Several epidemiological patterns are consistent across regions. Women have a substantially higher prevalence than men—approximately 50–70% greater [11–15, 57–59]—reflecting increased biological susceptibility to infection [60, 61]. Prevalence follows a clear gradient by sexual risk behavior, being highest among female sex workers and men who have sex with men, and lowest in the general population [11–15, 59]. Age-specific prevalence rises rapidly after sexual debut and largely plateaus by the mid-40s [11–15, 57–59]. There is also evidence of a cohort effect, with higher incidence observed in certain decades, such as in the United States [57, 62].

### Global Burden and Etiological Role of HSV-2 in Genital Ulcer Disease and Genital Herpes

Recent studies have reinforced the central role of HSV-2 in both clinically diagnosed genital ulcer disease and laboratory-confirmed genital herpes [1, 2, 11–15, 59]. In 2020, an estimated 187.9 million people aged 15–49 years experienced at least one episode of HSV-2-associated genital ulcer disease, corresponding to a global prevalence of 4.8% [1]. Prevalence was substantially higher in women (6.2%) than in men (3.5%) [1].

The proportion of genital ulcer disease attributable to HSV-2 mirrors its underlying prevalence [1, 2, 11–15]. The highest contribution is observed in sub-Saharan Africa, where HSV-2 accounts for more than half of all genital ulcer disease cases [11]. In Asia and in Latin America and the Caribbean, nearly half of genital ulcer disease cases are attributable to HSV-2 [12, 15], while in Australia, Europe, and Canada the proportion is lower, at around one-quarter [13, 14].

Globally, HSV-2 is the predominant cause of genital herpes [11–15], yet its contribution relative to herpes simplex virus type 1 (HSV-1) has declined in North America, Europe, and Australia over the past three decades [13, 14, 16]. This trend reflects reductions in HSV-2 prevalence in these regions [13, 14, 16, 57, 58], alongside the delay in the childhood acquisition rate of HSV-1 resulting in HSV-1 spread as an STI and a cause of genital herpes [16, 63–66].

### Global Burden and Clinical Impact of Neonatal Herpes

The principal concern with HSV-2 in pregnancy is preventing neonatal HSV infection, a condition associated with neurological injury, disseminated multi-organ infection, and long-term developmental disability [29, 30, 67]. Most cases (approximately 85%) are acquired perinatally through exposure to infected maternal genital secretions during delivery, with asymptomatic genital viral shedding—particularly during newly acquired maternal HSV infection—being the main route of transmission [29, 30, 68, 69].

Neonatal HSV infection is classified into three forms: skin, eye, and/or mouth; central nervous system; and disseminated disease [30, 67, 68]. Central nervous system and disseminated forms comprise roughly 30% and 25% of cases, respectively, with untreated case-fatality rates of up to 50% and 85% [29, 30, 68]. Management imposes substantial healthcare costs, including prolonged newborn hospitalization, intensive monitoring, intravenous antiviral therapy, and extensive diagnostic testing [29, 70–72]. Despite advances, mortality and long-term neurological morbidity remain high [29, 30, 67, 68, 72, 73].

Global mathematical modeling estimates the incidence rate of neonatal HSV infection, due to either HSV-2 or HSV-1, at around 10 cases per 100,000 live births [31]. Surveillance of this condition is limited and largely confined to regions within high-income settings [74]. A recent meta-analysis of data from high-income settings reported a pooled mean incidence rate of 8.2 per 100,000 live births, with an annual increase of approximately 3% [74]. The highest rates occur in the Americas, followed by Europe and the Western Pacific [74]. Globally, HSV-1 and HSV-2 each account for nearly half of all neonatal HSV infection cases, with marked regional variation in their relative contribution [74].

### Bidirectional Links between HSV-2 and HIV Infections: Epidemiology and Clinical Implications

Extensive observational evidence indicates that HSV-2 infection increases both the risk of acquiring and transmitting HIV infection, likely through mucosal disruption and chronic genital inflammation (Panel 1) [17–19]. A global meta-analysis found that, in general populations, prevalent HSV-2 infection was associated with a 2.7-fold higher risk of HIV acquisition, while incident HSV-2 infection was associated with a 4.7-fold higher risk [17]. HSV-2 may also increase HIV transmissibility by approximately 30% among persons not receiving antiretroviral therapy [18, 75].

In 2016, mathematical modeling estimated 1.4 million new sexually acquired HIV infections globally, including about 420,000 attributable to the synergy between HIV and HSV-2, with the greatest impact in Africa and among women [20]. However, randomized placebo-controlled trials of acyclovir suppressive therapy demonstrated no reduction in HIV acquisition or transmission, despite lowering HSV-2 shedding and disease recurrences [76–78].

HSV-2 prevalence is exceptionally high in people with HIV, driven by shared behavioral risk factors and probably biological interaction [79]. HSV-2 shedding and clinical reactivations are more frequent and severe among people with HIV reflecting compromised immunologic control [52–54]. In coinfected populations, suppressive antiviral therapy reduces HSV-2–related morbidity by decreasing the frequency and severity of symptomatic reactivations, limiting viral shedding, and, among those not on antiretroviral therapy, lowering plasma, rectal, and genital HIV RNA levels [80–83].

People with HIV with low CD4 T-cell counts and other immunocompromised persons are particularly prone to prolonged, severe, and sometimes atypical episodes of genital, perianal, or oral herpes [38, 84]. HSV-2 shedding is also increased in that population [85], and although antiretroviral therapy reduces the frequency and severity of HSV-2 recurrences, subclinical shedding remains frequent [53, 86]. Symptoms of genital herpes can also worsen during immune reconstitution after antiretroviral therapy initiation [86].

### Global Economic Burden of HSV-2

In 2016, global healthcare expenditures and productivity losses from HSV-2 infection were estimated at $31.2 billion U.S. dollars, with recurrent genital herpes episodes as the main cost [4]. The global economic burden of neonatal HSV infection attributable to HSV-2 was estimated at $53.4 million U.S. dollars in the same analysis [4].

In a separate study, across 90 low- and middle-income countries in 2019, genital herpes was estimated to cause $813.5 million U.S. dollars in treatment costs and productivity losses, rising to $29.0 billion U.S. dollars when accounting for care-seeking and absenteeism [5]. The quality-of-life impact was estimated at 61.7 million quality-adjusted life years lost, with mean annual costs per infection of $183 U.S. dollars for treatment and wage loss and $343.27 U.S. dollars for quality-adjusted life year loss [5].

### Diagnostic Methods for HSV-2

Accurate diagnosis of HSV-2 infection depends on the clinical context—whether active lesions are present—and on the use of appropriate laboratory methods [38, 87]. Virologic tests detect the virus directly from lesion swabs, whereas serologic assays detect antibodies indicating prior infection [38, 88, 89]. Table 1 summarizes the principal diagnostic methods, their performance, and indications.

**Table 1:** Common diagnostic tests for HSV-2 infection and their performance characteristics

Test	Typical Use Case	Sensitivity	Specificity	Comments
Polymerase chain reaction	Active genital lesions (preferred test)	90–100%	> 95%	• Detects viral DNA and distinguishes HSV-1 from HSV-2, with high sensitivity even during asymptomatic shedding.
Viral culture	Active lesions (if polymerase chain reaction unavailable)	Low to moderate sensitivity, particularly in recurrent lesions	~ 100% (high)	• Highly specific but limited in sensitivity, particularly for recurrent or healing lesions. • Enables antiviral resistance testing if viral growth is achieved.
Serologic Immunoglobulin G (type-specific)	Asymptomatic screening or diagnosis of past infection	~ 80–98% (after seroconversion)	80–95% (higher for high-index results; can be as low as 40% at low index)	• Detects lifelong antibodies to HSV-2. • False positives are common at low index values (1.1–3.0); low-positive results should be confirmed with a second assay (e.g., Western blot). • Not suitable for acute diagnosis, as seroconversion typically requires several weeks.
Western Blot (University of Washington)	Confirmation of HSV-2 serology	~ 99%	~ 100%	• Reference standard for HSV-2 antibody detection, performed in specialized laboratories. • Used to confirm equivocal or low-positive antibody serologic results.
HSV Immunoglobulin M serology	(Not recommended)	—	—	• Not type-specific, prone to false-positive results, and unable to reliably differentiate newly acquired from reactivating infection; therefore, not recommended for screening or diagnosis.

Abbreviations: *HSV-2*, herpes simplex virus type 2

#### Virologic Testing

For patients presenting with genital ulcers or mucocutaneous lesions consistent with HSV-2 infection, fluid and cellular material should be collected by swab for virologic confirmation [38]. Nucleic acid amplification testing, most commonly polymerase chain reaction, is the preferred diagnostic method [38]. Multiple U.S. Food and Drug Administration-approved assays exist. Polymerase chain reaction offers excellent sensitivity (90–100%) and specificity (> 95%) for detecting HSV DNA in lesion material, though performance may vary with assay design and primer selection [38, 90–92]. Importantly, polymerase chain reaction also differentiates HSV-2 from HSV-1, information essential for prognosis and counseling [38, 90–92].

If polymerase chain reaction is unavailable, viral culture can be performed; although highly specific when positive, its sensitivity is markedly lower—especially for recurrent or healing lesions [38, 93]. Antigen detection assays (e.g., direct fluorescent antibody tests) and cytology (Tzanck preparation) have low sensitivity and specificity, and are not recommended [38, 94]. In suspected HSV meningitis or neonatal HSV infection, polymerase chain reaction testing of cerebrospinal fluid or other relevant specimens remains the diagnostic standard [38, 95].

#### Serologic Testing

When lesions are absent, or to assess prior infection, HSV-type-specific serologic testing is recommended [38, 89]. Those assays detect immunoglobulin G (IgG) antibodies to HSV glycoprotein G1 or G2, enabling differentiation between prior HSV-1 and HSV-2 infection [38, 96, 97]. IgG antibodies typically develop within 12 weeks of infection and persist lifelong [38, 98].

Commercial HSV-2 IgG enzyme-linked immunosorbent assays, such as HerpeSelect, have reported sensitivities of approximately 80–98% for established infection [38, 99–103]. However, specificity is suboptimal, especially at low antibody index values. False positive results are frequent when the test index value fall within the low-positive range (e.g., 1.1–3.0), with specificity reported as low as 40% in this subgroup [38, 104–108]. In 2023, the U.S. Food and Drug Administration issued a Letter to Clinical Laboratory Staff and Healthcare Providers warning that HSV-2 serologic assays can yield false-positive results, particularly in persons with low pre-test probability or results close to the assay cutoff [109]. Those limitations translate into a low positive predictive value in general, low-prevalence populations, thereby reinforcing concerns about the feasibility and potential harms of general population-level screening.

Guidelines recommend confirmatory testing for low-index value positive HSV-2 serologic results before establishing a diagnosis [38]. Historically, confirmatory options included the Biokit HSV-2 Rapid Test (Biokit USA, Lexington, MA) and the University of Washington Western blot (Seattle, WA), the latter considered the gold standard, with near-100% sensitivity and specificity [38, 110, 111]. However, the Biokit assay is no longer commercially available, eliminating this option for confirmatory testing, and the Western blot is performed at a single reference laboratory, limiting accessibility. In the absence of confirmatory testing, patients should be counseled regarding the limitations and potential inaccuracy of a single low-index value positive result, and findings should be interpreted with caution in the context of the individual’s clinical presentation and pre-test probability of infection [38].

HSV immunoglobulin M (IgM) testing is not advised: IgM assays are not type-specific, frequently cross-react between HSV-1 and HSV-2, and cannot reliably distinguish recent from past infection as IgM can reappear during reactivation [38, 43].

#### Diagnostic Algorithms

Considering the available evidence on diagnostic methods, patients presenting with genital ulcers should undergo polymerase chain reaction testing with typing to differentiate HSV-1 from HSV-2 [38, 43]. If the polymerase chain reaction result is negative but clinical suspicion remains high—such as in healed or late-stage lesions—type-specific serology may be used to support the diagnosis of HSV-2 infection [38, 43]. However, a positive serologic result does not indicate how long the person has had the infection, and results may be falsely negative in recently acquired HSV infections [38, 43].

In asymptomatic persons without lesions, general population-level screening is not recommended; however, targeted screening may be appropriate in defined circumstances, such as for sex partners of persons with HSV-2 infection or persons at higher risk, including people with HIV, certain pregnant women, or those seeking sexual health care [37–45]. When serology is performed (e.g., at patient request or in groups at increased risk), type-specific IgG assays should be used, with confirmatory testing for positive results—particularly those in the low-positive range (index value 1.1–3.0)—to minimize incorrect diagnoses [38, 43]. Pre-test counseling is essential to explain the limitations of serology, including that a confirmed positive result reflects previously acquired infection without indicating time of acquisition or predicting symptom onset [38, 43]. Post-test counseling is equally important, particularly for those without prior symptoms, to address clinical and psychosocial implications [38, 43].

### Principles and Contemporary Frameworks for Screening

Screening policy is typically guided by the Wilson–Jungner principles, which require that the condition be a major health problem with effective interventions, adequate diagnostic and treatment capacity, and a detectable preclinical stage, using validated and acceptable tests within a clear intervention policy [112, 113]. Modern refinements emphasize equity, benefit–harm evaluation, cost-effectiveness modeling, informed choice, quality assurance, community engagement, robust data governance, and adaptation to local epidemiology and health system capacity [112–115].

### HSV-2 Screening: Balancing Potential Benefits against Proven Harms

Application of the Wilson–Jungner principles and contemporary screening frameworks [112–115] to HSV-2 highlights a central tension between potential benefits and well-recognized limitations and harms. It is important to emphasize that there is no controversy regarding diagnostic testing in symptomatic persons: confirming a diagnosis of genital herpes and identifying the viral type provides clear value for clinical management, counseling, and partner notification [38, 43]. The debate instead centers on screening—testing the general population of asymptomatic persons without signs or symptoms—and whether such an approach yields net benefit, and for which specific populations targeted screening might be justified.

#### Potential Benefits of Identifying Unrecognized Infection

Theoretical benefits of screening asymptomatic persons are plausible. Identifying previously unrecognized infection could enable patient education to promote safer sexual practices, such as consistent condom use and avoiding sex during periods of clinical symptoms; allow the offer of daily suppressive antiviral therapy to reduce viral shedding and transmission; and support partner notification so that partners can make informed choices or seek testing. In couples where one partner has HSV-2 infection, status awareness of the second partner is central to risk-reduction strategies. In pregnancy, knowledge of HSV-2 seropositivity could prompt heightened clinical vigilance during pregnancy and the period of delivery and the prophylactic use of suppressive antivirals [116–119].

#### Lack of Evidence for Population-level Benefit

Despite plausibility, no randomized trials have demonstrated that routine HSV-2 screening of asymptomatic persons reduces transmission or improves health outcomes [38, 120, 121]. Many HSV-2 antibody seropositive persons remain asymptomatic [6, 10, 48], limiting the potential benefit of antiviral therapy, and the effect of a diagnosis on the extent and durability of behavioral change to reduce partner transmission remains uncertain. Although daily valacyclovir reduces subclinical shedding [6, 122] and decreases the risk of transmission in HSV-2–discordant monogamous couples by 48% [123], evidence that it reduces population-level transmission is lacking [38]. In the absence of proven benefit—and considering the costs, adherence challenges, and psychosocial implications—treating asymptomatic persons is not currently justified as a general population-level strategy. Addressing these evidence gaps through well-designed studies could help clarify the potential value of screening and guide future policy recommendations.

#### Harms and Limitations of HSV-2 Screening

The potential harms of HSV-2 screening underpin recommendations against its routine use [37–44]. In low-prevalence populations (~ 10% prevalence), even tests with relatively high specificity have low positive predictive value; in the U.S., an assay with ~ 90% specificity can yield as many false-positive as true-positive results [102, 106, 111, 124, 125]. False-positive results can lead to substantial and avoidable anxiety [23, 126, 127]. Empirical evidence indicates that serologic diagnosis in asymptomatic individuals can induce transient psychological distress and herpes-specific quality-of-life impacts—particularly concerns regarding transmission, stigma, and intimate relationships—but without evidence of sustained or clinically significant psychological morbidity [23, 126, 127].

Accurate diagnoses also carry psychosocial burdens for both the person who transmitted and the person who acquired the infection, as genital herpes remains highly stigmatized [23–26]. Qualitative studies describe post-diagnosis depression, shame, fear of rejection, and difficulties with partner disclosure and sustaining relationships [23–26]. New diagnoses can also strain relationships, particularly when unexpected, fostering suspicion or eroding trust [23–26].

Although, from an individual patient perspective, there is a perceived “right to know” one’s infection status [128], for asymptomatic persons, screening confers a lifelong diagnosis without prior recognized illness, which may be viewed as unwarranted medicalization [129]. In the absence of a cure, prophylactic or therapeutic vaccine, or long-acting monoclonal antibodies—interventions that could alter disease course—management remains limited to suppressing symptoms and reducing recurrences, goals that have less relevance for people who are asymptomatic. Unlike hepatitis C virus infection [130], where screening can lead to curative treatment, the detection of HSV-2 infection offers no definitive resolution.

#### Ethical, Economic, and Other Considerations in HSV-2 Screening

The no-screening stance is not without debate. Proponents of sexual health autonomy argue that persons should be free to know their HSV-2 infection status to inform sexual activity and disclosure [129]. In practice, HSV-2 serology is frequently included in commercial “sexually transmitted disease (STD) panels” and direct-to-consumer tests [131]—often without adequate pre-test counseling—despite guidelines advising against routine use [37, 38, 109]. Expansion via telemedicine and at-home STI collection kits raises ethical concerns about result interpretation and whether exclusion of HSV-2 from such panels better serves the principle of non-maleficence [131]. However, persons seeking sexual health screening are often unaware that HSV-2 testing is not included and, if infected, may unknowingly place sex partners at risk.

Stigma and partner notification further complicate matters [129]. Cost-effectiveness analyses indicate that routine screening in the general population is not likely to be justified, given the costs of testing, confirmation, and management relative to uncertain benefit, with sexual health resources better directed toward interventions of proven population-level impact [132–134]. The cost per quality-adjusted life year gained exceeds accepted thresholds, as preventing a small number of neonatal HSV or HIV infection cases would require screening many thousands [132–134].

#### Balancing Benefits and Harms

Overall, the balance of evidence indicates that general population-level screening for HSV-2 is not justified. However, targeted screening—focused on groups at elevated risk or within specific contexts—may allow for patient autonomy and offer a more favorable balance of benefits to harms. This approach underpins current guidelines, which, despite differences in scope, consistently support individualized, context-specific screening strategies over universal population-level screening [37–45].

### HSV-2 Screening Guidelines across Health Systems

In the U.S., the U.S. Preventive Services Task Force advises against routine serologic screening for HSV-2 in asymptomatic adolescents and adults, including pregnant women, concluding with moderate certainty that potential harms outweigh benefits [37]. Key concerns include the low positive predictive value of current tests, psychosocial harm, missed detection of genital HSV-1 infection, absence of evidence for improved outcomes, and logistical barriers to confirmatory testing and counseling at scale [37, 125, 135, 136].

The Centers for Disease Control and Prevention recommends against HSV-2 serologic screening in asymptomatic persons and as part of routine prenatal care, while supporting a combination of targeted screening in specific contexts and diagnostic testing for those with symptoms [38]. Centers for Disease Control and Prevention guidelines emphasize identifying persons who are symptomatic or have a history suggestive of genital herpes and performing appropriate diagnostic testing in these cases [38]. The guidelines also acknowledge the role of targeted screening—such as among sex partners of persons with HSV-2 infection, people with HIV, or pregnant women at increased risk—where results would directly inform clinical management decisions [38]. Pregnancy management focuses on identifying women with active lesions or a history of genital herpes and preventing neonatal exposure, including the use of suppressive antiviral therapy in late pregnancy for those with recurrent disease [38, 116–119]. HSV-2 testing remains excluded from recommended STI screening panels for asymptomatic populations, including individuals seeking sexual healthcare [38].

In addition, the Centers for Disease Control and Prevention and the American College of Obstetricians and Gynecologists endorse targeted screening for pregnant women with sex partners known to have HSV-2 infection and for those with behaviors associated with increased STI or HIV risk, as well as diagnostic testing for individuals with recurrent or atypical genital symptoms but negative polymerase chain reaction or culture results [38, 39]. Preventive measures for HSV-2–discordant couples include counseling to avoid sexual contact during high-risk periods, particularly in the third-trimester of pregnancy [38, 39].

In Europe and elsewhere, a similar approach is followed. No country or international body recommends universal HSV-2 screening in asymptomatic populations [40–44]. The WHO likewise makes no recommendation for general population-based screening, instead emphasizing public education and integration of HSV-2 management into broader STI programs [137]. European and British Association for Sexual Health and HIV guidelines advise against serologic HSV-2 antibody screening in persons without symptoms, citing poor test specificity and lack of demonstrated benefit [40–44]. British Association for Sexual Health and HIV guidelines restricts the use of type-specific serology to defined indications, such as in those with recurrent atypical symptoms or having a sex partner with confirmed HSV-2 infection [43, 44]. Across guidelines, testing the asymptomatic partner in couples is supported to inform counseling on risk reduction—such as consistent condom use, avoidance of sexual contact during early symptoms or clinical recurrences, and daily suppressive antiviral therapy for the infected partner [37, 38, 40, 41, 43].

In people with HIV or other immunocompromised persons, particularly those with a history suggestive of prior genital herpes, type-specific serology can inform consideration of prophylactic antiviral therapy to prevent outbreaks (Panel 1) [37, 38, 40, 41]. According to the U.S. Adult and Adolescent Opportunistic Infections Guidelines, routine HSV-2 serologic screening is not recommended for people with HIV; however, type-specific serology may be considered for those with prior genital ulcer disease, atypical lesions not confirmed by polymerase chain reaction or culture, or an HSV-2–infected sex partner, with HSV-2 detection in a lesion and typing prioritized for symptomatic disease [45].

Panel 1 HSV-2 screening considerations in the context of HIV.

**Table:** 

•**Epidemiologic synergy** HSV-2 infection increases HIV acquisition risk two- to three-fold and enhances transmission by disrupting mucosa and sustaining genital inflammation. In 2016, an estimated 1.4 million people aged 15–49 years acquired HIV through sexual transmission, of whom about 420,000 cases were attributable to the synergy between HIV and HSV-2. HSV-2 prevalence is exceptionally high in people with HIV, reflecting both shared behavioral risks and biological interaction. • **Clinical course in people with HIV** HSV-2 shedding and reactivation are more frequent and severe in people with HIV, particularly with low CD4 + T-cell counts due to impaired immunologic control. Lesions are often painful, extensive, and atypical. Symptoms may transiently worsen during immune reconstitution after antiretroviral therapy initiation. Although antiretroviral therapy reduces recurrence frequency and severity, subclinical shedding remains common. • **Screening guidance** Despite the strong interaction, no international guideline recommends universal HSV-2 screening in people with HIV. However, type-specific serology may be considered at initial HIV evaluation, particularly in those with a history suggestive of genital herpes or in partnerships where one partner is known to be HSV-2 infected. Testing is also relevant for people with HIV with recurrent or atypical symptoms but negative HSV polymerase chain reaction test or culture, to inform diagnosis and potential suppressive therapy. • **Therapeutic implications** In people with HIV, HSV-2 diagnosis can guide suppressive antiviral therapy. Suppression reduces HSV-2 morbidity by decreasing outbreak frequency and severity, limiting viral shedding, and, among those not on antiretroviral therapy, lowering plasma, rectal, and genital HIV ribonucleic acid levels. Such treatment improves quality of life and may be particularly important in advanced immunosuppression to prevent severe herpes disease. • **Public health perspective** Randomized clinical trials of acyclovir in persons at risk for HIV or living with HIV have not demonstrated reductions in HIV acquisition or transmission. • **Future directions** Targeted screening strategies in high-incidence HIV settings remain a research priority. Advances such as highly specific antibody assays, novel antivirals, therapeutic vaccines, and curative therapies for HSV-2 infection may create opportunities to integrate HSV-2 screening into HIV prevention and care in the future.	

Abbreviations: HSV-2, herpes simplex virus type 2

The absence of routine screening across guidelines reflects a broad international consensus that, given the current diagnostic and therapeutic limitations, identifying those with asymptomatic HSV-2 infections is not a public health priority [37–45]. Exceptions are confined to situations where test results would directly influence clinical management, supporting a targeted screening approach [37–45]. In all cases, testing should be individualized and accompanied by pre- and post-test counseling [37, 38, 40, 41, 43].

### Putting the Pieces Together: Evidence, Constraints, and Emerging Technologies and Solutions for HSV-2 Screening

HSV-2 is a lifelong infection affecting more than half a billion people worldwide [1], with those living with infection shedding virus for roughly 40 days each year [6–8]—typically without recognizing their infectiousness—thereby sustaining transmission. That silent spread contributes to the substantial burden of disease, including ulcerative genital herpes, neonatal HSV infection, and other complications, along with persistent stigma, psychosocial distress, enhanced HIV transmission, and considerable economic costs. Although this profile makes screening for asymptomatic infection appear intuitively appealing, the current balance of evidence on benefits versus harms, combined with health system constraints, does not support routine serologic screening. Major barriers to screening include:

1. Limited proven benefit of current interventions

Screening would identify infections, but the vast majority of people living with HSV-2 remain asymptomatic or experience only mild disease. Available interventions—antiviral therapy and behavioral measures such as a reduction in the number of sex partners, avoidance of sex during symptoms, and condom use—have not been shown to translate the theoretical benefits of screening into measurable reductions in disease burden at the population level [37, 135]. Identification of infection rarely alters long-term outcomes for most persons. However, if future evidence demonstrates that suppressive or novel therapies in asymptomatic persons substantially reduces HSV-2 transmission or lowers HIV acquisition and transmission risk in high-incidence settings, the rationale for screening could be reconsidered.

Building evidence to support targeted screening requires data demonstrating clinical and public health value in defined populations. Future studies should assess diagnostic yield, predictive value, and clinical and behavioral outcomes among groups where results directly inform care—such as people with HIV, pregnant women with sex partners with HSV-2 infection, persons with recurrent or atypical symptoms, and in couples where one member has infection. Research should also evaluate how screening influences antiviral use, recurrence rates, partner transmission, and pregnancy management, while documenting psychosocial and economic outcomes. Pragmatic trials and modeling analyses that link test performance to actionable management pathways are essential to determine where targeted screening provides net benefit and to refine criteria for its implementation.

2. Diagnostic limitations

Most commercial serologic assays for HSV-2 infection have suboptimal specificity, with false positives common in low-prevalence populations and at low antibody index values. Confirmatory testing with the gold-standard Western blot is costly, technically complex, and restricted to specialized laboratories, limiting accessibility. An ideal solution would be a highly specific, affordable assay usable across diverse settings—including resource-limited clinics where a point-of-care assay would be helpful—that delivers same-visit results without the need for additional confirmation. Such tools could reduce loss to follow-up, facilitate timely counseling, and be valuable in environments such as STI clinics where counseling can occur and populations at increased risk for infection are seen.

Rapid and reliable diagnostics could also support selective strategies, such as integrating testing into care for people with HIV (Panel 1) or those with specific clinical presentations or behaviors. Improved tools would further enable an “opt-in” model, allowing patients—after counseling on benefits and harms—to choose testing, ensuring that positive results translate into clear management strategies while minimizing harm.

Emerging technologies, including Clustered Regularly Interspaced Short Palindromic Repeats -based diagnostics [138], show promise for sensitive, specific, and rapid detection of HSV-2 DNA without requiring typical laboratory infrastructure, and may supplement or even replace serologic testing in some contexts. If validated and scaled, these platforms could decentralize accurate diagnosis, support targeted screening approaches, and extend detection to subclinical shedding and active infection. Although still experimental, they illustrate the potential to transform the landscape of HSV-2 testing.

3. Need for biomedical innovation

The absence of transformative interventions remains the primary barrier to realizing the value of screening. Breakthroughs such as more potent antivirals, curative therapies, therapeutic vaccines, or targeted biologics could fundamentally alter this balance. Next-generation antivirals with enhanced suppressive efficacy could reduce HSV-2 transmission more effectively and potentially mitigate HIV acquisition and transmission risks [121, 139, 140]. Advances in gene editing [141] and promising vaccine candidates [35, 142–146] highlight the potential for tools that might either reduce the need for screening by lowering prevalence or be integrated into combined approaches, such as “screen-to-cure” or “screen-and-vaccinate” strategies. Sustained investment in these innovations is essential to make screening a meaningful public health intervention.

4. Psychosocial harms

An HSV-2 diagnosis in otherwise asymptomatic persons can result in profound psychosocial effects: stigma, anxiety, depression, fear of rejection, relationship strain, and disclosure difficulties [23–26]. These harms may be greater when diagnoses are unexpected or based on false-positive results [23–26]. However, available evidence suggests that such effects are generally transient, with no evidence of sustained or clinically significant psychological morbidity over time [23, 126, 127].

5. Changing epidemiology

An increasing proportion of genital herpes [13, 14, 16, 63–66] and neonatal HSV infection [74] is now attributable to HSV-1 infection, which is predominantly an oral infection [16, 63–66] and of lesser relevance for routine serologic detection in asymptomatic persons. Ideally, future interventions should target both HSV types and their associated disease burdens.

6. Logistical and resource constraints

Given the global and regional prevalence of HSV-2 infection [1, 11–15, 57–59], universal screening would require extensive infrastructure for testing and counseling millions of people—an unrealistic demand for most health systems.

### Targeted Screening and Diagnostic Testing

Although population-level screening for asymptomatic infection is not supported, diagnostic testing remains essential for symptomatic persons, where results directly inform clinical management. Targeted use of type-specific serology is also appropriate in well-defined situations where HSV-2 status influences care (Panel 2).

Panel 2 Indications for targeted HSV-2 serologic screening.

**Table:** 

Although population-level screening for asymptomatic infection is not supported, targeted type-specific serologic screening is appropriate in specific contexts: • **People with HIV or other immunocompromised persons**, specifically those with prior or suggestive symptoms of genital herpes, where type-specific serology can guide consideration of prophylactic antiviral therapy during immunosuppression. Screening may also be appropriate for those with atypical lesions not confirmed by polymerase chain reaction or culture, or with an HSV-2–infected partner, and can be considered at initial HIV evaluation based on individual risk profile. • **Pregnant women** whose partner has confirmed or suspected genital herpes, or partner is HSV-2–antibody seropositive, when the woman’s own serostatus is unknown, to guide preventive strategies and intrapartum management. • **HSV-2–infected persons in couples**, where status awareness informs risk-reduction counseling (e.g., condom use, avoidance of sex during outbreaks, daily suppressive therapy for the infected partner). • **Persons at higher epidemiologic risk**, such as those with multiple lifetime sex partners or presenting for STI evaluation. • **Persons with a clinical diagnosis** of genital herpes without laboratory confirmation. • **Persons with recurrent or atypical genital symptoms** but negative HSV polymerase chain reaction or culture results.	

Abbreviations: HSV-2, herpes simplex virus type 2

These include people with HIV or otherwise immunocompromised persons, particularly those with a history suggestive of genital herpes, with screening also considered during initial HIV evaluation (Panel 1). Pregnant women whose partner has confirmed or suspected genital herpes, or is HSV-2–seropositive, may benefit from screening when the woman’s own serostatus is unknown, as results can guide preventive strategies and intrapartum management. Screening is also relevant for couples where one partner is known to be infected, where awareness of infection status supports risk-reduction counseling—such as condom use, abstaining from sex, or changing sexual practices during outbreaks, and daily suppressive therapy for the infected partner.

Additional appropriate scenarios include persons at higher epidemiologic risk, such as those with multiple lifetime sex partners, persons with a clinical diagnosis of genital herpes without laboratory confirmation, and those with recurrent or atypical symptoms but negative polymerase chain reaction or culture results. In these contexts, targeted screening provides actionable information while limiting the potential harms of a new diagnosis.

## Conclusions and Way Forward

HSV-2 infection is one of the most prevalent STIs worldwide, affecting nearly one in eight adults [1]. Despite this burden, routine general population-level serologic screening of asymptomatic persons is not currently justified. Evidence shows no demonstrable population-level benefit, while the risks of false-positive diagnoses, stigma, and psychosocial harm exist—although greater public awareness and testing may help reduce stigma. Clinical and public health practice has therefore emphasized accurate diagnostic testing in symptomatic persons and targeted screening in well-defined groups where results meaningfully inform care. This stratified approach maximizes benefit while minimizing harm, reflecting a consistent international consensus reaffirmed by recent policy updates and regulatory advisories on test limitations, although its implementation in practice remains variable and inconsistent.

Looking forward, emerging technologies, including rapid point-of-care assays, hold promise to improve test accuracy, accessibility, and timeliness. Parallel advances in more potent antivirals, therapeutic vaccines, and curative strategies could eventually alter the harm-cost–benefit calculus of screening, potentially enabling approaches such as “screen-to-cure” or “screen-and-vaccinate”. Until such breakthroughs occur, however, the foundation of HSV-2 infection control lies in utilizing evidence-based diagnostic testing, targeted screening, patient education, destigmatization, and preventive measures to reduce transmission. HSV-2 screening exemplifies that more testing is not always better; the priority is to direct interventions where they achieve the most benefit with the least harm, while remaining prepared for innovations that may transform future practice.

### Limitations

This review has limitations. First, the synthesis relies primarily on published evidence, existing guidelines, and policy documents, which are themselves constrained by gaps in high-quality data on HSV-2 screening. Few randomized trials or large-scale prospective studies have evaluated the effectiveness of screening asymptomatic persons, leaving many recommendations dependent on indirect evidence, modeling studies, or expert consensus.

Second, the review emphasizes high-income country guidelines (e.g., U.S. Preventive Services Task Force, Centers for Disease Control and Prevention, European, and United Kingdom guidance). Evidence and practice patterns from low- and middle-income countries, where HSV-2 prevalence is often higher and health system capacity differs, are underrepresented due to the limited availability of relevant published literature. This may restrict the generalizability of conclusions across diverse global contexts.

Third, the rapidly evolving fields of diagnostics, antivirals, curative therapies, and vaccine development introduce inherent uncertainty. Emerging tools—such as point-of-care assays, gene-editing approaches, and therapeutic vaccines—show promise but their clinical utility, accessibility, and cost-effectiveness remain unproven. Accordingly, some forward-looking statements in this review should be regarded as provisional, if not speculative, and contingent on future validation.

Finally, the psychosocial and ethical dimensions of HSV-2 infection screening are complex and unevenly studied. Available evidence highlights stigma and psychological harm, but these outcomes are difficult to quantify and likely vary across cultural and social settings. The conclusions of this overview should therefore be interpreted with caution, recognizing that further empirical research—particularly from high HSV-2 and HIV infection burden, resource-limited settings—is needed to refine evidence-based policies on HSV-2 infection screening.

## Key References

- Harfouche M, AlMukdad S, Alareeki A, Osman AMM, Gottlieb S, Rowley J, et al. Estimated global and regional incidence and prevalence of herpes simplex virus infections and genital ulcer disease in 2020: mathematical modelling analyses. Sex Transm Infect. 2025;101(4):214-23. doi: 10.1136/sextrans-2024–056307.
  - ◦The paper presents the latest World Health Organization global and regional estimates of the incidence and prevalence of HSV infections and genital ulcer disease for 2020.
- Workowski KA, Bachmann LH, Chan PA, Johnston CM, Muzny CA, Park I, et al. Sexually Transmitted Infections Treatment Guidelines, 2021. MMWR Recomm Rep. 2021;70(4):1–187. doi: 10.15585/mmwr.rr7004a1.
  - ◦The paper presents the 2021 U.S. Centers for Disease Control and Prevention (CDC) Sexually Transmitted Infections Treatment Guidelines, offering comprehensive, evidence-based recommendations for the diagnosis, management, treatment, and prevention of HSV-2 infection.
- Patel R, Moran B, Clarke E, Geretti AM, Lautenschlager S, Green J, et al. 2024 European guidelines for the management of genital herpes. J Eur Acad Dermatol Venereol. 2025;39(4):742-58. doi: 10.1111/jdv.20450.
  - ◦The paper presents the 2024 European Guidelines for the Management of Genital Herpes, developed by the European STI Guidelines Editorial Board, and provides updated, evidence-based recommendations for the diagnosis, management, treatment, and prevention of HSV-2 infection.
- Patel R, Green J, Moran B, Clarke E, Seneviratne K, Evans C, et al. British Association of Sexual Health and HIV UK national guideline for the management of anogenital herpes, 2024. Int J STD AIDS. 2025;36(2):90–105. doi: 10.1177/09564624241282396.
  - ◦The paper presents the 2024 UK National Guideline for the Management of Anogenital Herpes, developed by the British Association for Sexual Health and HIV (BASHH), and provides updated, evidence-based recommendations for the diagnosis, management, treatment, and prevention of HSV-2 infection.

## Data Availability

No datasets were generated or analyzed during the current study.

## References

[1] Harfouche M, AlMukdad S, Alareeki A, Osman AMM, Gottlieb S, Rowley J, et al. Estimated global and regional incidence and prevalence of herpes simplex virus infections and genital ulcer disease in 2020: mathematical modelling analyses. Sex Transm Infect. 2025;101(4):214–23. 10.1136/sextrans-2024-056307. 39658199 10.1136/sextrans-2024-056307 PMC12128767

[2] Looker KJ, Johnston C, Welton NJ, James C, Vickerman P, Turner KME, et al. The global and regional burden of genital ulcer disease due to herpes simplex virus: a natural history modelling study. BMJ Glob Health. 2020;5(3):e001875. 10.1136/bmjgh-2019-001875. 32201620 10.1136/bmjgh-2019-001875 PMC7061890

[3] Johnston C, Wald A. Genital Herpes. JAMA. 2024;332(10):835–6. 10.1001/jama.2024.12743. 39141407 10.1001/jama.2024.12743

[4] Chaiyakunapruk N, Lee SWH, Kulchaitanaroaj P, Rayanakorn A, Lee H, Looker KJ, et al. Estimated global and regional economic burden of genital herpes simplex virus infection among 15–49 year-olds in 2016. BMC Glob Public Health. 2024;2(1):42. 10.1186/s44263-024-00053-6. 39681948 10.1186/s44263-024-00053-6 PMC11618196

[5] Silva S, Ayoub HH, Johnston C, Atun R, Abu-Raddad LJ. Estimated economic burden of genital herpes and HIV attributable to herpes simplex virus type 2 infections in 90 low- and middle-income countries: a modeling study. PLoS Med. 2022;19(12):e1003938. 10.1371/journal.pmed.1003938. 36520853 10.1371/journal.pmed.1003938 PMC9754187

[6] Tronstein E, Johnston C, Huang ML, Selke S, Magaret A, Warren T, et al. Genital shedding of herpes simplex virus among symptomatic and asymptomatic persons with HSV-2 infection. JAMA. 2011;305(14):1441–9. 10.1001/jama.2011.420. 21486977 10.1001/jama.2011.420 PMC3144252

[7] Mark KE, Wald A, Magaret AS, Selke S, Olin L, Huang ML, et al. Rapidly cleared episodes of herpes simplex virus reactivation in immunocompetent adults. J Infect Dis. 2008;198(8):1141–9. 10.1086/591913. 18783315 10.1086/591913 PMC2667115

[8] Schiffer JT, Abu-Raddad L, Mark KE, Zhu J, Selke S, Magaret A, et al. Frequent release of low amounts of herpes simplex virus from neurons: results of a mathematical model. Sci Transl Med. 2009;1(7):7ra16. 10.1126/scitranslmed.3000193. 20161655 10.1126/scitranslmed.3000193 PMC2818652

[9] Langenberg AG, Corey L, Ashley RL, Leong WP, Straus SE, Chiron HSV Vaccine Study Group. A prospective study of new infections with herpes simplex virus type 1 and type 2. N Engl J Med. 1999;341(19):1432–8. 10.1056/NEJM199911043411904. 10547406 10.1056/NEJM199911043411904

[10] Wald A, Zeh J, Selke S, Warren T, Ryncarz AJ, Ashley R, et al. Reactivation of genital herpes simplex virus type 2 infection in asymptomatic seropositive persons. N Engl J Med. 2000;342(12):844–50. 10.1056/NEJM200003233421203. 10727588 10.1056/NEJM200003233421203

[11] Harfouche M, Abu-Hijleh FM, James C, Looker KJ, Abu-Raddad LJ. Epidemiology of herpes simplex virus type 2 in sub-Saharan Africa: systematic review, meta-analyses, and meta-regressions. EClinicalMedicine. 2021;35:100876. 10.1016/j.eclinm.2021.100876. 34027335 10.1016/j.eclinm.2021.100876 PMC8129943

[12] AlMukdad S, Harfouche M, Wettstein A, Abu-Raddad LJ. Epidemiology of herpes simplex virus type 2 in Asia: a systematic review, meta-analysis, and meta-regression. Lancet Reg Health West Pac. 2021;12:100176. 10.1016/j.lanwpc.2021.100176. 34527970 10.1016/j.lanwpc.2021.100176 PMC8356094

[13] Alareeki A, Osman AMM, Khandakji MN, Looker KJ, Harfouche M, Abu-Raddad LJ. Epidemiology of herpes simplex virus type 2 in Europe: systematic review, meta-analyses, and meta-regressions. The Lancet Regional Health - Europe. 2023;25:100558. 10.1016/j.lanepe.2022.100558. 36818238 10.1016/j.lanepe.2022.100558 PMC9929610

[14] AlMukdad S, Farooqui US, Harfouche M, Aldos L, Abu-Raddad LJ. Epidemiology of herpes simplex virus type 2 in Canada, Australia, and New Zealand: systematic review, meta-analyses, and meta-regressions. Sex Transm Dis. 2022;49(6):403–13. 10.1097/OLQ.0000000000001612. 35608096 10.1097/OLQ.0000000000001612 PMC9093718

[15] Harfouche M, Maalmi H, Abu-Raddad LJ. Epidemiology of herpes simplex virus type 2 in Latin America and the Caribbean: systematic review, meta-analyses and metaregressions. Sex Transm Infect. 2021;97(7):490–500. 10.1136/sextrans-2021-054972. 34088792 10.1136/sextrans-2021-054972 PMC8543241

[16] Ageeb RA, Harfouche M, Chemaitelly H, Abu-Raddad LJ. Epidemiology of herpes simplex virus type 1 in the United States: systematic review, meta-analyses, and meta-regressions. iScience. 2024;27(9):110652. 10.1016/j.isci.2024.110652. 39224512 10.1016/j.isci.2024.110652 PMC11367537

[17] Looker KJ, Elmes JAR, Gottlieb SL, Schiffer JT, Vickerman P, Turner KME, et al. Effect of HSV-2 infection on subsequent HIV acquisition: an updated systematic review and meta-analysis. Lancet Infect Dis. 2017;17(12):1303–16. 10.1016/S1473-3099(17)30405-X. 28843576 10.1016/S1473-3099(17)30405-X PMC5700807

[18] Abu-Raddad LJ, Magaret AS, Celum C, Wald A, Longini IM Jr., Self SG, et al. Genital herpes has played a more important role than any other sexually transmitted infection in driving HIV prevalence in Africa. PloS one. 2008;3(5):e2230. 10.1371/journal.pone.0002230. 18493617 10.1371/journal.pone.0002230 PMC2377333

[19] Freeman EE, Weiss HA, Glynn JR, Cross PL, Whitworth JA, Hayes RJ. Herpes simplex virus 2 infection increases HIV acquisition in men and women: systematic review and meta-analysis of longitudinal studies. AIDS. 2006;20(1):73–83. 16327322 10.1097/01.aids.0000198081.09337.a7

[20] Looker KJ, Welton NJ, Sabin KM, Dalal S, Vickerman P, Turner KME, et al. Global and regional estimates of the contribution of herpes simplex virus type 2 infection to HIV incidence: a population attributable fraction analysis using published epidemiological data. Lancet Infect Dis. 2020;20(2):240–9. 10.1016/S1473-3099(19)30470-0. 31753763 10.1016/S1473-3099(19)30470-0 PMC6990396

[21] Omori R, Nagelkerke N, Abu-Raddad LJ. HIV and herpes simplex virus type 2 epidemiological synergy: misguided observational evidence? A modelling study. Sex Transm Infect. 2018;94(5):372–6. 10.1136/sextrans-2017-053336. 29203577 10.1136/sextrans-2017-053336 PMC6204970

[22] Gupta R, Warren T, Wald A. Genital herpes. Lancet. 2007;370(9605):2127–37. 10.1016/S0140-6736(07)61908-4. 18156035 10.1016/S0140-6736(07)61908-4

[23] Melville J, Sniffen S, Crosby R, Salazar L, Whittington W, Dithmer-Schreck D, et al. Psychosocial impact of serological diagnosis of herpes simplex virus type 2: a qualitative assessment. Sex Transm Infect. 2003;79(4):280–5. 10.1136/sti.79.4.280. 12902574 10.1136/sti.79.4.280 PMC1744709

[24] Wang K, Merin A, Rendina HJ, Pachankis JE. Genital herpes stigma: toward the measurement and validation of a highly prevalent yet hidden public health problem. Stigma Health. 2018;3(1):27–34. 10.1037/sah0000067. 29629409 10.1037/sah0000067 PMC5881938

[25] Fraley SS. Psychosocial outcomes in individuals living with genital herpes. J Obstet Gynecol Neonatal Nurs. 2002;31(5):508–13. 10.1111/j.1552-6909.2002.tb00074.x. 12353728 10.1111/j.1552-6909.2002.tb00074.x

[26] Mark H, Gilbert L, Nanda J. Psychosocial well-being and quality of life among women newly diagnosed with genital herpes. J Obstet Gynecol Neonatal Nurs. 2009;38(3):320–6. 10.1111/j.1552-6909.2009.01026.x. 19538620 10.1111/j.1552-6909.2009.01026.x

[27] Fisman DN. Health related quality of life in genital herpes: a pilot comparison of measures. Sex Transm Infect. 2005;81(3):267–70. 10.1136/sti.2004.011619. 15923300 10.1136/sti.2004.011619 PMC1744978

[28] Mindel A, Marks C. Psychological symptoms associated with genital herpes virus infections: epidemiology and approaches to management. CNS Drugs. 2005;19(4):303–12. 10.2165/00023210-200519040-00003. 15813644 10.2165/00023210-200519040-00003

[29] Corey L, Wald A. Maternal and neonatal herpes simplex virus infections. N Engl J Med. 2009;361(14):1376–85. 10.1056/NEJMra0807633. 19797284 10.1056/NEJMra0807633 PMC2780322

[30] Samies NL, James SH, Kimberlin DW. Neonatal herpes simplex virus disease: updates and continued challenges. Clin Perinatol. 2021;48(2):263–74. 10.1016/j.clp.2021.03.003. 34030813 10.1016/j.clp.2021.03.003

[31] Looker KJ, Magaret AS, May MT, Turner KME, Vickerman P, Newman LM, et al. First estimates of the global and regional incidence of neonatal herpes infection. Lancet Glob Health. 2017;5(3):e300–9. 10.1016/S2214-109X(16)30362-X. 28153513 10.1016/S2214-109X(16)30362-X PMC5837040

[32] Pinninti SG, Kimberlin DW. Maternal and neonatal herpes simplex virus infections. Am J Perinatol. 2013;30(2):113–9. 10.1055/s-0032-1332802. 23303485 10.1055/s-0032-1332802

[33] Gottlieb SL, Deal CD, Giersing B, Rees H, Bolan G, Johnston C, et al. The global roadmap for advancing development of vaccines against sexually transmitted infections: update and next steps. Vaccine. 2016;34(26):2939–47. 10.1016/j.vaccine.2016.03.111. 27105564 10.1016/j.vaccine.2016.03.111 PMC6759054

[34] Gottlieb SL, Spielman E, Abu-Raddad L, Aderoba AK, Bachmann LH, Blondeel K, et al. WHO global research priorities for sexually transmitted infections. Lancet Glob Health. 2024;12(9):e1544–51. 10.1016/S2214-109X(24)00266-3. 39043199 10.1016/S2214-109X(24)00266-3 PMC11342064

[35] Johnston C, Scheele S, Bachmann L, Boily MC, Chaiyakunapruk N, Deal C, et al. Vaccine value profile for herpes simplex virus. Vaccine. 2024;42(19S1):S82–100. 10.1016/j.vaccine.2024.01.044. 39003018 10.1016/j.vaccine.2024.01.044

[36] U. S. Department of Health Human Services. HSV Addendum to the Sexually Transmitted Infections National Strategic Plan for the United States. 2021–2025. Available from: https://www.hhs.gov/sites/default/files/hsv-addendum-national-strategic-plan.pdf. Accessed on: December 28, 2025. US Department of Health and Human Services. 2024.

[37] Asher GN, Feltner C, Harrison WN, Schwimmer E, Rains C, Jonas DE. Serologic screening for genital herpes: updated evidence report and systematic review for the US preventive services task force. JAMA. 2023;329(6):510–2. 10.1001/jama.2022.20356. 36786798 10.1001/jama.2022.20356 PMC9929693

[38] Workowski KA, Bachmann LH, Chan PA, Johnston CM, Muzny CA, Park I, et al. Sexually transmitted infections treatment guidelines, 2021. MMWR Recomm Rep. 2021;70(4):1–187. 10.15585/mmwr.rr7004a1. 34292926 10.15585/mmwr.rr7004a1 PMC8344968

[39] American College of Obstetricians and Gynecologists. Management of genital herpes in pregnancy: ACOG practice Bulletin. ACOG practice Bulletin, number 220. Obstet Gynecol. 2020;135(5):e193–202. 10.1097/AOG.0000000000003840. 32332414 10.1097/AOG.0000000000003840

[40] Patel R, Kennedy OJ, Clarke E, Geretti A, Nilsen A, Lautenschlager S, et al. 2017 European guidelines for the management of genital herpes. Int J STD AIDS. 2017;28(14):1366–79. 10.1177/0956462417727194. 28836892 10.1177/0956462417727194

[41] Patel R, Moran B, Clarke E, Geretti AM, Lautenschlager S, Green J, et al. 2024 European guidelines for the management of genital herpes. J Eur Acad Dermatol Venereol. 2025;39(4):742–58. 10.1111/jdv.20450. 39620271 10.1111/jdv.20450 PMC11934026

[42] Patel R, Green J, Clarke E, Seneviratne K, Abbt N, Evans C, et al. 2014 UK National guideline for the management of anogenital herpes. Int J STD AIDS. 2015;26(11):763–76. 10.1177/0956462415580512. 25861804 10.1177/0956462415580512

[43] Patel R, Green J, Moran B, Clarke E, Seneviratne K, Evans C, et al. British Association of Sexual Health and HIV UK national guideline for the management of anogenital herpes, 2024. Int J STD AIDS. 2025;36(2):90–105. 10.1177/09564624241282396. 39374063 10.1177/09564624241282396 PMC11773994

[44] British Association of Sexual Health and HIV Clinical Effectiveness Group. 2023 BASHH Summary Guidance on Testing for Sexually Transmitted Infections. Available from: https://www.bashh.org/_userfiles/pages/files/resources/bashh_summary_guidance_on_stis_testing_2023.pdf?utm_source=chatgpt.com. Accessed on: October 25, 2025. British Association for Sexual Health and HIV. 2023.

[45] National Institutes of Health, Centers for Disease Control and Prevention, and Infectious Diseases Society of America. Guidelines for the Prevention and Treatment of Opportunistic Infections in Adults and Adolescents with HIV: Herpes Simplex Virus. Available from: https://clinicalinfo.hiv.gov/en/guidelines/hiv-clinical-guidelines-adult-and-adolescent-opportunistic-infections/herpes-simplex. Accessed on: October 25, 2025. National Institutes of Health, Centers for Disease Control and Prevention, and Infectious Diseases Society of America. 2024.

[46] Phipps W, Nakku-Joloba E, Krantz EM, Selke S, Huang ML, Kambugu F, et al. Genital herpes simplex virus type 2 shedding among adults with and without HIV infection in Uganda. J Infect Dis. 2016;213(3):439–47. 10.1093/infdis/jiv451. 26486633 10.1093/infdis/jiv451 PMC4704673

[47] Benedetti J, Corey L, Ashley R. Recurrence rates in genital herpes after symptomatic first-episode infection. Ann Intern Med. 1994;121(11):847–54. 7978697 10.7326/0003-4819-121-11-199412010-00004

[48] Langenberg A, Benedetti J, Jenkins J, Ashley R, Winter C, Corey L. Development of clinically recognizable genital lesions among women previously identified as having “asymptomatic” herpes simplex virus type 2 infection. Ann Intern Med. 1989;110(11):882–7. 10.7326/0003-4819-110-11-882. 2541645 10.7326/0003-4819-110-11-882

[49] Corey L, Langenberg AG, Ashley R, Sekulovich RE, Izu AE, Douglas JM Jr., et al. Recombinant glycoprotein vaccine for the prevention of genital HSV-2 infection: two randomized controlled trials. Chiron HSV vaccine study group. JAMA. 1999;282(4):331–40. 10.1001/jama.282.4.331. 10432030 10.1001/jama.282.4.331

[50] Phipps W, Saracino M, Magaret A, Selke S, Remington M, Huang ML, et al. Persistent genital herpes simplex virus-2 shedding years following the first clinical episode. J Infect Dis. 2011;203(2):180–7. 10.1093/infdis/jiq035. 21288817 10.1093/infdis/jiq035 PMC3058740

[51] Schiffer JT, Corey L. Rapid host immune response and viral dynamics in herpes simplex virus-2 infection. Nat Med. 2013;19(3):280–90. 10.1038/nm.3103. 23467247 10.1038/nm.3103 PMC3981536

[52] Posavad CM, Koelle DM, Shaughnessy MF, Corey L. Severe genital herpes infections in HIV-infected individuals with impaired herpes simplex virus-specific CD8 + cytotoxic T lymphocyte responses. Proc Natl Acad Sci U S A. 1997;94(19):10289–94. 10.1073/pnas.94.19.10289. 9294203 10.1073/pnas.94.19.10289 PMC23355

[53] Posavad CM, Wald A, Kuntz S, Huang ML, Selke S, Krantz E, et al. Frequent reactivation of herpes simplex virus among HIV-1-infected patients treated with highly active antiretroviral therapy. J Infect Dis. 2004;190(4):693–6. 10.1086/422755. 15272395 10.1086/422755

[54] Schiffer JT, Swan DA, Magaret A, Schacker TW, Wald A, Corey L. Mathematical modeling predicts that increased HSV-2 shedding in HIV-1 infected persons is due to poor immunologic control in ganglia and genital mucosa. PLoS One. 2016;11(6):e0155124. 10.1371/journal.pone.0155124. 27285483 10.1371/journal.pone.0155124 PMC4902308

[55] Magaret AS, Mujugira A, Hughes JP, Lingappa J, Bukusi EA, DeBruyn G, et al. Effect of condom use on per-act HSV-2 transmission risk in HIV-1, HSV-2-discordant couples. Clin Infect Dis. 2016;62(4):456–61. 10.1093/cid/civ908. 26578538 10.1093/cid/civ908 PMC4725379

[56] Schiffer JT, Mayer BT, Fong Y, Swan DA, Wald A. Herpes simplex virus-2 transmission probability estimates based on quantity of viral shedding. J R Soc Interface. 2014;11(95):20140160. 10.1098/rsif.2014.0160. 24671939 10.1098/rsif.2014.0160 PMC4006256

[57] Ayoub HH, Amara I, Awad SF, Omori R, Chemaitelly H, Abu-Raddad LJ. Analytic characterization of the herpes simplex virus type 2 epidemic in the United States, 1950–2050. Open Forum Infect Dis. 2021;8(7):ofab218. 10.1093/ofid/ofab218. 34262986 10.1093/ofid/ofab218 PMC8274361

[58] Chemaitelly H, Nagelkerke N, Omori R, Abu-Raddad LJ. Characterizing herpes simplex virus type 1 and type 2 seroprevalence declines and epidemiological association in the United States. PLoS One. 2019;14(6):e0214151. 10.1371/journal.pone.0214151. 31170140 10.1371/journal.pone.0214151 PMC6553692

[59] Harfouche M, Alareeki A, Osman AMM, Alaama AS, Hermez JG, Abu-Raddad LJ. Epidemiology of herpes simplex virus type 2 in the Middle East and North Africa: systematic review, meta-analyses, and meta-regressions. J Med Virol. 2023;95(3):e28603. 10.1002/jmv.28603. 36815489 10.1002/jmv.28603

[60] Hachem H, Ayoub HH, Abu-raddad LJ. Sex-Specific Biological Susceptibility and Sexual Mixing Patterns in Herpes Simplex Virus Type 2 Transmission. under review. 2025. 10.1186/s44263-026-00270-1 PMC13107675 42026694

[61] Wald A, Corey L. Persistence in the population: epidemiology, transmission. In: Arvin A, Campadelli-Fiume G, Mocarski E, Moore PS, Roizman B, Whitley R editors Human Herpesviruses: Biology, Therapy, and Immunoprophylaxis. Cambridge University Press. 2007;36: 656–674.

[62] Naffeti BS, Ayoub HH, Abu-Raddad LJ. Quantifying population-level sexual risk behavior through HSV-2 transmission dynamics in the United States, 1950–2020. Sci Rep. 2025;15(1):34521. 10.1038/s41598-025-17719-2. 41044103 10.1038/s41598-025-17719-2 PMC12494987

[63] AlMukdad S, Harfouche M, Farooqui US, Aldos L, Abu-Raddad LJ. Epidemiology of herpes simplex virus type 1 and genital herpes in Australia and New Zealand: systematic review, meta-analyses and meta-regressions. Epidemiol Infect. 2023;151:e33. 10.1017/S0950268823000183. 36750224 10.1017/S0950268823000183 PMC9990408

[64] AlMukdad S, Harfouche M, Farooqui US, Aldos L, Abu-Raddad LJ. Epidemiology of herpes simplex virus type 1 in Canada: systematic review, meta-analyses, and meta-regressions. Front Public Health. 2023;11:1118249. 10.3389/fpubh.2023.1118249. 37521995 10.3389/fpubh.2023.1118249 PMC10375289

[65] Yousuf W, Ibrahim H, Harfouche M, Abu Hijleh F, Abu-Raddad L. Herpes simplex virus type 1 in Europe: systematic review, meta-analyses and meta-regressions. BMJ Glob Health. 2020. 10.1136/bmjgh-2020-002388. 32675066 10.1136/bmjgh-2020-002388 PMC7369148

[66] Ayoub HH, Chemaitelly H, Abu-Raddad LJ. Characterizing the transitioning epidemiology of herpes simplex virus type 1 in the USA: model-based predictions. BMC Med. 2019;17(1):57. 10.1186/s12916-019-1285-x. 30853029 10.1186/s12916-019-1285-x PMC6410528

[67] James SH, Kimberlin DW. Neonatal herpes simplex virus infection: epidemiology and treatment. Clin Perinatol. 2015;42(1):47–59. 10.1016/j.clp.2014.10.005. 25677996 10.1016/j.clp.2014.10.005

[68] James SH, Kimberlin DW. Neonatal herpes simplex virus infection. Infect Dis Clin North Am. 2015;29(3):391–400. 10.1016/j.idc.2015.05.001. 26154662 10.1016/j.idc.2015.05.001

[69] Arvin AM. Relationships between maternal immunity to herpes simplex virus and the risk of neonatal herpesvirus infection. Rev Infect Dis. 1991;13(Suppl 11):S953–6. 10.1093/clind/13.supplement_11.s953. 1664133 10.1093/clind/13.supplement_11.s953

[70] Ambroggio L, Lorch SA, Mohamad Z, Mossey J, Shah SS. Congenital anomalies and resource utilization in neonates infected with herpes simplex virus. Sex Transm Dis. 2009;36(11):680–5. 10.1097/OLQ.0b013e3181aaf54f. 19617865 10.1097/OLQ.0b013e3181aaf54f PMC2783783

[71] Flagg EW, Weinstock H. Incidence of neonatal herpes simplex virus infections in the united States, 2006. Pediatrics. 2011;127(1):e1–8. 10.1542/peds.2010-0134. 21149432 10.1542/peds.2010-0134

[72] Melvin AJ, Mohan KM, Vora SB, Selke S, Sullivan E, Wald A. Neonatal herpes simplex virus infection: epidemiology and outcomes in the modern era. J Pediatr Infect Dis Soc. 2022;11(3):94–101. 10.1093/jpids/piab105. 10.1093/jpids/piab105 PMC8946680 34894240

[73] Kimberlin DW. Why neonatal herpes matters. Lancet Glob Health. 2017;5(3):e234–5. 10.1016/S2214-109X(17)30047-5. 28153515 10.1016/S2214-109X(17)30047-5

[74] Saed Aldien A, Harfouche M, Alareeki A, Chemaitelly H, Abu-Raddad LJ. Global epidemiology of neonatal herpes: systematic review, meta-analyses, and meta-regressions. Under review. 2025. 10.7189/jogh.16.04104 PMC13003892 41860333

[75] Silhol R, Coupland H, Baggaley RF, Miller L, Staadegaard L, Gottlieb SL, et al. What is the burden of heterosexually acquired HIV due to HSV-2? Global and regional model-based estimates of the proportion and number of HIV infections attributable to HSV-2 infection. J Acquir Immune Defic Syndr. 2021;88(1):19–30. 10.1097/QAI.0000000000002743. 34117163 10.1097/QAI.0000000000002743 PMC8397258

[76] Celum C, Wald A, Lingappa JR, Magaret AS, Wang RS, Mugo N, et al. Acyclovir and transmission of HIV-1 from persons infected with HIV-1 and HSV-2. N Engl J Med. 2010;362(5):427–39. 10.1056/NEJMoa0904849. 20089951 10.1056/NEJMoa0904849 PMC2838503

[77] Watson-Jones D, Weiss HA, Rusizoka M, Changalucha J, Baisley K, Mugeye K, et al. Effect of herpes simplex suppression on incidence of HIV among women in Tanzania. N Engl J Med. 2008;358(15):1560–71. 10.1056/NEJMoa0800260. 18337596 10.1056/NEJMoa0800260 PMC2643126

[78] Celum C, Wald A, Hughes J, Sanchez J, Reid S, Delany-Moretlwe S, et al. Effect of aciclovir on HIV-1 acquisition in herpes simplex virus 2 seropositive women and men who have sex with men: a randomised, double-blind, placebo-controlled trial. Lancet. 2008;371(9630):2109–19. 10.1016/S0140-6736(08)60920-4. 18572080 10.1016/S0140-6736(08)60920-4 PMC2650104

[79] Kouyoumjian SP, Heijnen M, Chaabna K, Mumtaz GR, Omori R, Vickerman P, et al. Global population-level association between herpes simplex virus 2 prevalence and HIV prevalence. AIDS. 2018;32(10):1343–52. 10.1097/QAD.0000000000001828. 29794495 10.1097/QAD.0000000000001828 PMC5991180

[80] Baeten JM, Strick LB, Lucchetti A, Whittington WL, Sanchez J, Coombs RW, et al. Herpes simplex virus (HSV)-suppressive therapy decreases plasma and genital HIV-1 levels in HSV-2/HIV-1 coinfected women: a randomized, placebo-controlled, cross-over trial. J Infect Dis. 2008;198(12):1804–8. 10.1086/593214. 18928378 10.1086/593214 PMC2665183

[81] Zuckerman RA, Lucchetti A, Whittington WL, Sanchez J, Coombs RW, Zuniga R, et al. Herpes simplex virus (HSV) suppression with valacyclovir reduces rectal and blood plasma HIV-1 levels in HIV-1/HSV-2-seropositive men: a randomized, double-blind, placebo-controlled crossover trial. J Infect Dis. 2007;196(10):1500–8. 10.1086/522523. 18008230 10.1086/522523

[82] Delany S, Mlaba N, Clayton T, Akpomiemie G, Capovilla A, Legoff J, et al. Impact of aciclovir on genital and plasma HIV-1 RNA in HSV-2/HIV-1 co-infected women: a randomized placebo-controlled trial in South Africa. AIDS. 2009;23(4):461–9. 10.1097/QAD.0b013e32831db217. 19155993 10.1097/QAD.0b013e32831db217 PMC2948532

[83] Nagot N, Ouedraogo A, Foulongne V, Konate I, Weiss HA, Vergne L, et al. Reduction of HIV-1 RNA levels with therapy to suppress herpes simplex virus. N Engl J Med. 2007;356(8):790–9. 10.1056/NEJMoa062607. 17314338 10.1056/NEJMoa062607

[84] Leeyaphan C, Surawan TM, Chirachanakul P, Prasertworonun N, Punyaratabandhu P, Omcharoen V, et al. Clinical characteristics of hypertrophic herpes simplex genitalis and treatment outcomes of imiquimod: a retrospective observational study. Int J Infect Dis. 2015;33:165–70. 10.1016/j.ijid.2015.02.002. 25660091 10.1016/j.ijid.2015.02.002

[85] Keller MJ, Huber A, Espinoza L, Serrano MG, Parikh HI, Buck GA, et al. Impact of herpes simplex virus type 2 and human immunodeficiency virus dual infection on female genital tract mucosal immunity and the vaginal microbiome. J Infect Dis. 2019;220(5):852–61. 10.1093/infdis/jiz203. 31111902 10.1093/infdis/jiz203 PMC6667798

[86] Tobian AA, Grabowski MK, Serwadda D, Newell K, Ssebbowa P, Franco V, et al. Reactivation of herpes simplex virus type 2 after initiation of antiretroviral therapy. J Infect Dis. 2013;208(5):839–46. 10.1093/infdis/jit252. 23812240 10.1093/infdis/jit252 PMC3733512

[87] Ashley-Morrow R, Krantz E, Wald A. Time course of seroconversion by herpeselect ELISA after acquisition of genital herpes simplex virus type 1 (HSV-1) or HSV-2. Sex Transm Dis. 2003;30(4):310–4. 10.1097/00007435-200304000-00007. 12671550 10.1097/00007435-200304000-00007

[88] Scoular A. Using the evidence base on genital herpes: optimising the use of diagnostic tests and information provision. Sex Transm Infect. 2002;78(3):160–5. 10.1136/sti.78.3.160. 12238644 10.1136/sti.78.3.160 PMC1744455

[89] Wald A, Ashley-Morrow R. Serological testing for herpes simplex virus (HSV)-1 and HSV-2 infection. Clin Infect Dis. 2002;35(Suppl 2):S173–82. 10.1086/342104. 12353203 10.1086/342104

[90] Binnicker MJ, Espy MJ, Duresko B, Irish C, Mandrekar J. Automated processing, extraction and detection of herpes simplex virus types 1 and 2: a comparative evaluation of three commercial platforms using clinical specimens. J Clin Virol. 2017;89:30–3. 10.1016/j.jcv.2017.02.006. 28226272 10.1016/j.jcv.2017.02.006

[91] Teo JW, Chiang D, Jureen R, Lin RT. Clinical evaluation of a helicase-dependant amplification (HDA)-based commercial assay for the simultaneous detection of HSV-1 and HSV-2. Diagn Microbiol Infect Dis. 2015;83(3):261–2. 10.1016/j.diagmicrobio.2015.07.018. 26302856 10.1016/j.diagmicrobio.2015.07.018

[92] Gitman MR, Ferguson D, Landry ML. Comparison of simplexa HSV 1 & 2 PCR with culture, immunofluorescence, and laboratory-developed TaqMan PCR for detection of herpes simplex virus in swab specimens. J Clin Microbiol. 2013;51(11):3765–9. 10.1128/JCM.01413-13. 24006008 10.1128/JCM.01413-13 PMC3889765

[93] Wald A, Huang ML, Carrell D, Selke S, Corey L. Polymerase chain reaction for detection of herpes simplex virus (HSV) DNA on mucosal surfaces: comparison with HSV isolation in cell culture. J Infect Dis. 2003;188(9):1345–51. 10.1086/379043. 14593592 10.1086/379043

[94] Caviness AC, Oelze LL, Saz UE, Greer JM, Demmler-Harrison GJ. Direct immunofluorescence assay compared to cell culture for the diagnosis of mucocutaneous herpes simplex virus infections in children. J Clin Virol. 2010;49(1):58–60. 10.1016/j.jcv.2010.06.006. 20620099 10.1016/j.jcv.2010.06.006

[95] Lyons TW, Cruz AT, Freedman SB, Nigrovic LE, Herpes Simplex Virus Study Group of the Pediatric Emergency Medicine Collaborative Research C. Accuracy of herpes simplex virus polymerase chain reaction testing of the blood for central nervous system herpes simplex virus infections in infants. J Pediatr. 2018;200:274-6 e1. 10.1016/j.jpeds.2018.04.061. 29784511 10.1016/j.jpeds.2018.04.061

[96] Ashley RL. Genital herpes. Type-specific antibodies for diagnosis and management. Dermatol Clin. 1998;16(4):789–93. 10.1016/s0733-8635(05)70048-6. 9891682 10.1016/s0733-8635(05)70048-6

[97] Ashley RL. Performance and use of HSV type-specific serology test kits. Herpes. 2002;9(2):38–45. 12106510

[98] Ashley R, Benedetti J, Corey L. Humoral immune response to HSV-1 and HSV-2 viral proteins in patients with primary genital herpes. J Med Virol. 1985;17(2):153–66. 10.1002/jmv.1890170208. 2997384 10.1002/jmv.1890170208

[99] Whittington WL, Celum CL, Cent A, Ashley RL. Use of a glycoprotein G-based type-specific assay to detect antibodies to herpes simplex virus type 2 among persons attending sexually transmitted disease clinics. Sex Transm Dis. 2001;28(2):99–104. 10.1097/00007435-200102000-00007. 11234793 10.1097/00007435-200102000-00007

[100] Turner KR, Wong EH, Kent CK, Klausner JD. Serologic herpes testing in the real world: validation of new type-specific serologic herpes simplex virus tests in a public health laboratory. Sex Transm Dis. 2002;29(7):422–5. 10.1097/00007435-200207000-00011. 12170133 10.1097/00007435-200207000-00011

[101] Eing BR, Lippelt L, Lorentzen EU, Hafezi W, Schlumberger W, Steinhagen K, et al. Evaluation of confirmatory strategies for detection of type-specific antibodies against herpes simplex virus type 2. J Clin Microbiol. 2002;40(2):407–13. 11825950 10.1128/JCM.40.2.407-413.2002 PMC153348

[102] Ashley-Morrow R, Nollkamper J, Robinson NJ, Bishop N, Smith J. Performance of focus ELISA tests for herpes simplex virus type 1 (HSV-1) and HSV-2 antibodies among women in ten diverse geographical locations. Clin Microbiol Infect. 2004;10(6):530–6. 10.1111/j.1469-0691.2004.00836.x. 15191381 10.1111/j.1469-0691.2004.00836.x

[103] Morrow RA, Friedrich D, Krantz E. Performance of the focus and Kalon enzyme-linked immunosorbent assays for antibodies to herpes simplex virus type 2 glycoprotein G in culture-documented cases of genital herpes. J Clin Microbiol. 2003;41(11):5212–4. 14605166 10.1128/JCM.41.11.5212-5214.2003 PMC262548

[104] Ngo TD, Laeyendecker O, La H, Hogrefe W, Morrow RA, Quinn TC. Use of commercial enzyme immunoassays to detect antibodies to the herpes simplex virus type 2 glycoprotein G in a low-risk population in Hanoi, Vietnam. Clin Vaccine Immunol. 2008;15(2):382–4. 10.1128/CVI.00437-06. 18077617 10.1128/CVI.00437-06 PMC2238064

[105] Agyemang E, Le QA, Warren T, Magaret AS, Selke S, Johnston C, et al. Performance of Commercial Enzyme-Linked Immunoassays for Diagnosis of Herpes Simplex Virus-1 and Herpes Simplex Virus-2 Infection in a Clinical Setting. Sex Transm Dis. 2017;44(12):763–7. 10.1097/OLQ.0000000000000689. 28876290 10.1097/OLQ.0000000000000689

[106] Golden MR, Ashley-Morrow R, Swenson P, Hogrefe WR, Handsfield HH, Wald A. Herpes simplex virus type 2 (HSV-2) western blot confirmatory testing among men testing positive for HSV-2 using the focus enzyme-linked immunosorbent assay in a sexually transmitted disease clinic. Sex Transm Dis. 2005;32(12):771–7. 10.1097/01.olq.0000175377.88358.f3. 16314775 10.1097/01.olq.0000175377.88358.f3

[107] Biraro S, Mayaud P, Morrow RA, Grosskurth H, Weiss HA. Performance of commercial herpes simplex virus type-2 antibody tests using serum samples from Sub-Saharan Africa: a systematic review and meta-analysis. Sex Transm Dis. 2011;38(2):140–7. 10.1097/OLQ.0b013e3181f0bafb. 20706175 10.1097/OLQ.0b013e3181f0bafb

[108] Dargham SR, Nasrallah GK, Al-Absi ES, Mohammed LI, Al-Disi RS, Nofal MY, et al. Herpes Simplex Virus Type 2 Seroprevalence Among Different National Populations of Middle East and North African Men. Sex Transm Dis. 2018;45(7):482–7. 10.1097/OLQ.0000000000000791. 29465656 10.1097/OLQ.0000000000000791 PMC6012054

[109] Food US, Administration D. HSV-2 Serological Tests Can Produce False Positive Results: Letter to Clinical Laboratory Staff and Health Care Providers. Available from: https://www.fda.gov/medical-devices/letters-health-care-providers/hsv-2-tests-genital-herpes-can-produce-false-reactive-results-letter-clinical-laboratory-staff-and. Accessed on: August 14, 2025. U.S. Food and Drug Administration; 2023.

[110] Morrow R, Friedrich D. Performance of a novel test for IgM and IgG antibodies in subjects with culture-documented genital herpes simplex virus-1 or -2 infection. Clin Microbiol Infect. 2006;12(5):463–9. 10.1111/j.1469-0691.2006.01370.x. 16643524 10.1111/j.1469-0691.2006.01370.x

[111] Morrow RA, Friedrich D, Meier A, Corey L. Use of biokit HSV-2 rapid assay to improve the positive predictive value of focus herpeselect HSV-2 ELISA. BMC Infect Dis. 2005;5:84. 10.1186/1471-2334-5-84. 16225691 10.1186/1471-2334-5-84 PMC1276011

[112] Dobrow MJ, Hagens V, Chafe R, Sullivan T, Rabeneck L. Consolidated principles for screening based on a systematic review and consensus process. CMAJ. 2018;190(14):E422–9. 10.1503/cmaj.171154. 29632037 10.1503/cmaj.171154 PMC5893317

[113] Sturdy S, Miller F, Hogarth S, Armstrong N, Chakraborty P, Cressman C, et al. Half a century of Wilson & Jungner: reflections on the governance of population screening. Wellcome Open Res. 2020;5:158. 10.12688/wellcomeopenres.16057.2. 32923689 10.12688/wellcomeopenres.16057.1 PMC7468564

[114] Harris R, Sawaya GF, Moyer VA, Calonge N. Reconsidering the criteria for evaluating proposed screening programs: reflections from 4 current and former members of the U.S. preventive services task force. Epidemiol Rev. 2011;33:20–35. 10.1093/epirev/mxr005. 21666224 10.1093/epirev/mxr005

[115] Andermann A, Blancquaert I, Beauchamp S, Dery V. Revisiting Wilson and Jungner in the genomic age: a review of screening criteria over the past 40 years. Bull World Health Organ. 2008;86(4):317–9. 10.2471/blt.07.050112. 18438522 10.2471/BLT.07.050112 PMC2647421

[116] Watts DH, Brown ZA, Money D, Selke S, Huang ML, Sacks SL, et al. A double-blind, randomized, placebo-controlled trial of acyclovir in late pregnancy for the reduction of herpes simplex virus shedding and cesarean delivery. Am J Obstet Gynecol. 2003;188(3):836–43. 10.1067/mob.2003.185. 12634667 10.1067/mob.2003.185

[117] Scott LL, Hollier LM, McIntire D, Sanchez PJ, Jackson GL, Wendel GD Jr. Acyclovir suppression to prevent recurrent genital herpes at delivery. Infect Dis Obstet Gynecol. 2002;10(2):71–7. 10.1155/S1064744902000054. 12530483 10.1155/S1064744902000054 PMC1784606

[118] Braig S, Luton D, Sibony O, Edlinger C, Boissinot C, Blot P, et al. Acyclovir prophylaxis in late pregnancy prevents recurrent genital herpes and viral shedding. Eur J Obstet Gynecol Reprod Biol. 2001;96(1):55–8. 10.1016/s0301-2115(00)00406-1. 11311761 10.1016/s0301-2115(00)00406-1

[119] Sheffield JS, Hill JB, Hollier LM, Laibl VR, Roberts SW, Sanchez PJ, et al. Valacyclovir prophylaxis to prevent recurrent herpes at delivery: a randomized clinical trial. Obstet Gynecol. 2006;108(1):141–7. 10.1097/01.AOG.0000219749.96274.15. 16816068 10.1097/01.AOG.0000219749.96274.15

[120] Owen EM, Jama M, Nahal B, Clarke E, Obasi A. 20 years of herpes simplex virus type 2 (HSV-2) research in low-income and middle-income countries: systematic evaluation of progress made in addressing WHO priorities for research in HSV-2/HIV interactions, HSV-2 control and mathematical modelling. BMJ Glob Health. 2024. 10.1136/bmjgh-2024-015167. 38964882 10.1136/bmjgh-2024-015167 PMC11227757

[121] Johnston C. Diagnosis and management of genital herpes: key questions and review of the evidence for the 2021 centers for disease control and prevention sexually transmitted infections treatment guidelines. Clin Infect Dis. 2022;74(Suppl2):S134–43. 10.1093/cid/ciab1056. 35416970 10.1093/cid/ciab1056

[122] Martens MG, Fife KH, Leone PA, Dix LP, Brennan CA. Once daily valacyclovir for reducing viral shedding in subjects newly diagnosed with genital herpes. Infect Dis Obstet Gynecol. 2009;2009:105376. 10.1155/2009/105376. 19680456 10.1155/2009/105376 PMC2724712

[123] Corey L, Wald A, Patel R, Sacks SL, Tyring SK, Warren T, et al. Once-daily valacyclovir to reduce the risk of transmission of genital herpes. N Engl J Med. 2004;350(1):11–20. 10.1056/NEJMoa035144. 14702423 10.1056/NEJMoa035144

[124] Mark HD, Nanda JP, Roberts J, Rompalo A, Melendez JH, Zenilman J. Performance of focus ELISA tests for HSV-1 and HSV-2 antibodies among university students with no history of genital herpes. Sex Transm Dis. 2007;34(9):681–5. 10.1097/01.olq.0000258307.18831.f0. 17457239 10.1097/01.olq.0000258307.18831.f0 PMC2648390

[125] Feltner C, Grodensky CA, Ebel C, Jackson GR, Woodell CL, Coker-Schwimmer EJL, et al. Serologic screening for genital herpes infection: an evidence review for the U.S. Preventive services task Force. evidence Syntheses, no 149. Rockville (MD): Agency for Healthcare Research and Quality, U.S. Department of Health and Human Services;; 2016.

[126] Rosenthal SL, Zimet GD, Leichliter JS, Stanberry LR, Fife KH, Tu W, et al. The psychosocial impact of serological diagnosis of asymptomatic herpes simplex virus type 2 infection. Sex Transm Infect. 2006;82(2):154–7. 10.1136/sti.2005.016311. 16581745 10.1136/sti.2005.016311 PMC2564691

[127] Meyer JL, Crosby RA, Whittington WL, Carrell D, Ashley-Morrow R, Meier AS, et al. The psychosocial impact of serological herpes simplex type 2 testing in an urban HIV clinic. Sex Transm Infect. 2005;81(4):309–15. 10.1136/sti.2004.012146. 16061537 10.1136/sti.2004.012146 PMC1745021

[128] Beauchamp TL, Childress JF. Principles of biomedical ethics. Eighth edition. ed. New York: Oxford University Press; 2019.

[129] Krantz I, Lowhagen GB, Ahlberg BM, Nilstun T. Ethics of screening for asymptomatic herpes virus type 2 infection. BMJ. 2004;329(7466):618–21. 10.1136/bmj.329.7466.618. 15361449 10.1136/bmj.329.7466.618 PMC516665

[130] Bhattacharya D, Aronsohn A, Price J, Lo Re V, AASLD-IDSA HCV Guidance Panel. Hepatitis C guidance 2023 update: AASLD-IDSA recommendations for Testing, Managing, and treating hepatitis C virus infection. Clin Infect Dis. 2023. 10.1093/cid/ciad319. 37229695 10.1093/cid/ciad319

[131] Exten C, Pinto CN, Gaynor AM, Meyerson B, Griner SB, Van Der Pol B. Direct-to-consumer sexually transmitted infection testing services: a position statement from the American Sexually Transmitted Diseases Association. Sex Transm Dis. 2021;48(11):e155–9. 10.1097/OLQ.0000000000001475. 34030157 10.1097/OLQ.0000000000001475 PMC8505153

[132] Fisman DN, Hook EW 3rd, Goldie SJ. Estimating the costs and benefits of screening monogamous, heterosexual couples for unrecognised infection with herpes simplex virus type 2. Sex Transm Infect. 2003;79(1):45–52. 10.1136/sti.79.1.45. 12576614 10.1136/sti.79.1.45 PMC1744602

[133] Thung SF, Grobman WA. The cost-effectiveness of routine antenatal screening for maternal herpes simplex virus-1 and – 2 antibodies. Am J Obstet Gynecol. 2005;192(2):483–8. 10.1016/j.ajog.2004.09.134. 15695991 10.1016/j.ajog.2004.09.134

[134] Little S, Kaimal A, Caughey A. 217: antenatal screening for HSV: the cost-effectiveness of various screening modalities. Am J Obstet Gynecol. 2007;197(6):S72. 10.1016/j.ajog.2007.10.230.17825653

[135] Pearlman MD. Reducing HSV-2 morbidity and mortality: routine serologic screening still not the best answer. JAMA. 2023;329(6):469–71. 10.1001/jama.2022.24870. 36786801 10.1001/jama.2022.24870

[136] Bibbins-Domingo K, Grossman DC, Curry SJ, Davidson KW, Epling JW Jr., et al. Serologic screening for genital herpes infection: US Preventive Services Task Force recommendation statement. JAMA. 2016;316(23):2525–30. 10.1001/jama.2016.16776. 27997659 10.1001/jama.2016.16776

[137] World Health Organization. Global health sector strategies on, respectively, HIV, viral hepatitis and sexually transmitted infections for the period 2022–2030: WHO policy brief. Available from: https://www.who.int/publications/i/item/9789240053779. Accessed on: August 15, 2025. Geneva, Switzerland: World Health Organization; 2022.

[138] Yin X, Luo H, Zhou H, Zhang Z, Lan Y, Feng Z, et al. A rapid isothermal CRISPR-Cas13a diagnostic test for genital herpes simplex virus infection. iScience. 2024;27(1):108581. 10.1016/j.isci.2023.108581. 38213624 10.1016/j.isci.2023.108581 PMC10783623

[139] Birkmann A, Saunders R. Overview on the management of herpes simplex virus infections: current therapies and future directions. Antiviral Res. 2025;237:106152. 10.1016/j.antiviral.2025.106152. 40154924 10.1016/j.antiviral.2025.106152

[140] Ellison ST, Hayman I, Derr K, Derr P, Frebert S, Itkin Z, et al. Limitations of acyclovir and identification of potent HSV antivirals using 3D bioprinted human skin equivalents. Nat Commun. 2025;16(1):9200. 10.1038/s41467-025-64245-w. 41102222 10.1038/s41467-025-64245-w PMC12533247

[141] Aubert M, Haick AK, Strongin DE, Klouser LM, Loprieno MA, Stensland L, et al. Gene editing for latent herpes simplex virus infection reduces viral load and shedding in vivo. Nat Commun. 2024;15(1):4018. 10.1038/s41467-024-47940-y. 38740820 10.1038/s41467-024-47940-y PMC11091195

[142] Johnston C, Gottlieb SL, Wald A. Status of vaccine research and development of vaccines for herpes simplex virus. Vaccine. 2016;34(26):2948–52. 10.1016/j.vaccine.2015.12.076. 26973067 10.1016/j.vaccine.2015.12.076

[143] Bernstein DI, Wald A, Warren T, Fife K, Tyring S, Lee P, et al. Therapeutic vaccine for genital herpes simplex Virus-2 infection: findings from a randomized trial. J Infect Dis. 2017;215(6):856–64. 10.1093/infdis/jix004. 28329211 10.1093/infdis/jix004 PMC7206854

[144] Van Wagoner N, Fife K, Leone PA, Bernstein DI, Warren T, Panther L, et al. Effects of different doses of GEN-003, a therapeutic vaccine for genital herpes simplex Virus-2, on viral shedding and lesions: results of a randomized Placebo-Controlled trial. J Infect Dis. 2018;218(12):1890–9. 10.1093/infdis/jiy415. 29982727 10.1093/infdis/jiy415 PMC7191615

[145] Gottlieb SL, Giersing BK, Hickling J, Jones R, Deal C, Kaslow DC, et al. Meeting report: initial world health organization consultation on herpes simplex virus (HSV) vaccine preferred product characteristics, March 2017. Vaccine. 2019;37(50):7408–18. 10.1016/j.vaccine.2017.10.084. 29224963 10.1016/j.vaccine.2017.10.084

[146] Quadiri A, Prakash S, Dhanushkodi NR, Singer M, Zayou L, Shaik AM, et al. Therapeutic prime/pull vaccination of HSV-2-infected Guinea pigs with the ribonucleotide reductase 2 (RR2) protein and CXCL11 chemokine boosts antiviral local tissue-resident and effector memory CD4(+) and CD8(+) T cells and protects against recurrent genital herpes. J Virol. 2024;98(5):e0159623. 10.1128/jvi.01596-23. 38587378 10.1128/jvi.01596-23 PMC11092353

